# In contrast to matrix metalloproteinases, serum adiponectin concentrations increase after radioiodine treatment of thyrotoxicosis

**DOI:** 10.1186/1756-6614-5-12

**Published:** 2012-10-29

**Authors:** A Lewiński, A Brona, KC Lewandowski, E Skowrońska-Jóźwiak, A Milewicz

**Affiliations:** 1 Department of Endocrinology and Metabolic Diseases, Medical University of Lodz, Rzgowska St. No. 281/289, 93-338 Lodz, Poland; 2 Department of Endocrinology, Diabetes and Isotope Therapy, Medical University of Wroclaw, Wroclaw, Poland

**Keywords:** Matrix metalloproteinases, Adiponectin, Thyrotoxicosis, Radioactive iodine

## Abstract

**Background:**

Matrix metalloproteinases (MMPs), together with their tissue inhibitors (TIMPs), remodel extracellular matrix under physiological and pathological conditions and are implicated in pathogenesis of cardiovascular diseases, cancer and in chronic inflammation. We have endeavoured to assess whether concentrations of MMPs, TIMPs, and anti-inflammatory adiponectin are altered by pharmacological treatment of acute thyrotoxicosis or by radioiodine therapy (RIT).

**Material and methods:**

We measured serum concentrations of MMP-2, MMP-9, TIMP-1, TIMP-2, and adiponectin, TSH, free T_4_ (FT_4_) and free T_3_ (FT_3_) in 15 patients (4 males), age (years) 51.8±15.3 (mean±SD) with hyperthyroidism treated with thiamazole (Group 1) and in 20 subjects (2 males), treated for thyrotoxicosis with radioiodine, age 52.3±12.4 (Group 2), where blood samples were taken before RIT, visit 1 (V1), seven days post RIT, visit 2 (V2), and two to three months post RIT, visit 3 (V3).

**Results:**

In Group 1 there was no significant change in concentrations of MMP-2, MMP-9, TIMP-1, TIMP-2 or adiponectin, despite a fall in FT_4_ and FT_3_ (8.74±4.79 pg/ml vs 3.54±2.40 pg/ml, for FT_3_, and 4.48 ±2.21 ng/ml vs 1.02±1.07 ng/ml, for FT_4_, p<0.001). In Group 2 RIT did not cause any acute change in serum MMP-2, MMP-9, TIMP-1 and TIMP-2 or adiponectin (V1 vs V2). However, there was a significant increase in serum adiponectin [from 15201±8860 ng/ml (V1) to 19373±8657 ng/ml (at V3), p<0.05], and TIMP-2 at V3 [from 129±45 ng/ml (V1) to 149±38 ng/ml (V3), p<0.01]. There was no significant change MMP-2, MMP-9 and TIMP-1 between V1 and V3. There was a decrease in FT_4_ and FT_3_ from 24.4±15.4 pmol/l (V1) to 14.7±10.6 pmol/l (V3), and from 10.0±5.65 (V1) to 6.1±4.8 pmol/l (V2), p<0.01, for FT_4_ and FT_3_, respectively.

**Conclusions:**

Radioiodine therapy of thyrotoxicosis does not alter serum MMP-2, MMP-9 or TIMP-1 concentrations either acutely or after about three months of observation. An increase in serum adiponectin might reflect favourable effects of radioiodine administration on cardiovascular risk factors, while an increase in TIMP-2 (principal MMP-2 inhibitor) might lead to a decrease in free MMP-2 concentrations.

## Introduction

The term matrix metalloproteinases (MMPs) refers to a group of enzymes that remodel extracellular matrix in various physiological and pathological conditions, such as neoplasms, inflammatory and cardiovascular diseases [1]. In particular, increased activity of MMPs in blood vessels has been implicated in formation of aneurysms [2], as well as in formation of unstable atherosclerotic plaques, in turn, leading to an increased risk of thrombotic and embolic events, including myocardial infarctions and strokes [3-5].

Adiponectin is an abundant plasma protein secreted from adipocytes. It has been thought to be a key molecule in the development of type 2 diabetes mellitus and metabolic syndrome, which are epidemiological targets for preventing cardiovascular disease. In addition to beneficial metabolic effects, adiponectin seems to have anti-inflammatory, anti-atherosclerotic and vasoprotective actions. Furthermore, adiponectin affects signaling in myocardial cells and exerts beneficial actions on the heart after pressure overload and ischemia-reperfusion injury [6,7].

There are reports [8,9], suggesting that even subclinical thyrotoxicosis may be independently associated, with an increased cardiovascular morbidity and mortality, though there is no universal agreement on this subject [10]. However, in contrast to thyroid neoplasms, concentrations of MMPs during treatment of thyrotoxicosis have not been assessed, so far.

Pharmacological treatment of thyrotoxicosis carries a definite risk of agranulocytosis that cannot be prevented by routine complete blood count (CBC) monitoring of asymptomatic patients [11]. Furthermore, pharmacological treatment generally does not lead to permanent cure in subjects with toxic multinodular goitre, and is associated with relapse in over 50% of cases of Graves’s disease. For these reasons radioactive iodine treatment (RIT) has been used for many years to treat thyrotoxicosis. That treatment was considered to be safe enough, to be prescribed even for children with Graves’ disease [12]. There are, however, some data [13] suggestive that RIT might lead to an increase in cardiovascular and cancer mortality at least in some subjects, though a definite causality remains to be proven [14]. Given potential option of surgery (though also associated with risks of complications), the safety of RIT is of paramount importance from the clinical view-point. For these reasons, we have endeavoured to investigate whether serum concentrations of MMPs, their inhibitors (TIMP-1, TIMP-2) change during treatment with radioiodine. For comparison we have also investigated whether treatment of thyrotoxicosis *per se* might be also associated with changes of serum concentrations of MMPs, TIMPs and adiponectin.

## Subjects and methods

The study involved two groups of subjects: Group 1 – patients with thyrotoxicosis before and after treatment with thiamazole, and Group 2 – patients with thyrotoxicosis, who underwent therapy with radioactive iodine. We studied 15 patients (4 males), age 51.8±15.3 years (mean±SD), BMI 24.7±3.5 kg/m^2^_,_ with hyperthyroidism due to Graves’ disease (n=8), toxic adenoma (n=1) or toxic multinodular goitre (n=6), treated with thiamazole. Thyroid function, including concentrations of TSH, free T_4_ (FT_4_) and free T_3_ (FT_3_), was assessed before treatment and after 4–8 weeks. In all these subjects we also measured concentration of matrix metalloproteinase 2 (MMP-2), matrix metalloproteinase-9 (MMP-9), tissue inhibitor of matrix metalloproteinases-1 (TIMP-1), tissue inhibitor of matrix metalloproteinases-2 (TIMP-2) and adiponectin, before and after treatment with thiamazole.

Group 2 included 20 subjects (2 males), treated for thyrotoxicosis with radioiodine, age 52.3±12.4 years, BMI 27.4±4.66 kg/m^2^, where blood samples were taken before RIT, visit 1 (V1), seven days post RIT, visit 2 (V2), and two to three months post RIT, visit 3 (V3). The etiology of thyrotoxicosis included Graves’ disease (n=10), toxic adenoma (n=3) or multinodular goitre (n=7). Radioactive iodine was administered according to the protocol that involved thyroid goitre or nodule volume, radioiodine uptake (T24) and radioiodine activity for 1 gram of thyroid tissue in the gland (depending on thyroid disease, see below). The formula for calculation the dose of radioiodine was as follows:

dose of radioiodine (mCi) = [thyroid weight (g)^1^ x radioiodine activity for 1 g of thyroid tissue (μCi/g)^2^] / [(T24 (%)^3^ × 10],

where:

1. 1 ml of thyroid volume means 1 g of thyroid tissue,

2. radioiodine activity to be administered in adults: in Graves’ disease - 80–150 μCi/1 g of thyroid tissue, in toxic thyroid nodule - 150 μCi/1 g of thyroid tissue, in toxic nodular goitre - 100–150 μCi/1 g of thyroid tissue,

3. radioiodine uptake in % [15].

Measurements of MMP-2, MMP-9, TIMP-1, TIMP-2 and adiponectin were performed by R & D systems immunoassays (Human Quantikine ELISA kit, for MMPs and TIMPs, Human Total Adiponectin /Acrp30 Quantikine ELISA Kit - cataloque numbers: DMP2F0 for MMP–2, DMP900 for MMP9, DTM100 for TIMP1, DTM200 for TIMP-2 and DRP300 for adiponectin). Measurements of FT_4_, FT_3_ and TSH were performed in Group 1 by the means of Elecsys electrochemiluminescent immunoassay (Roche® Diagnostics GmbH), while in Group 2 they were performed by the means of Immulite 2000 XPi Immunoassay System (Siemens AG Germany).

### Statistical analysis

The data were analysed by means of simple descriptive statistics of location and dispersion and Friedman ANOVA for dependent samples (repeated measurements). If the observed difference between all measurements was significant, *post hoc* Tukey’s test was performed. In case of non-normal distribution of data Mann-Whitney’s test for a comparison of pairs of measurements. In all analyses, statistical significance was considered achieved at a value of p ≤ 0.05. All the calculations were derived by means of Statistica v9.0 software.

The study was approved by the Ethics Committee of the Medical University of Lodz, Poland.

## Results

**Figure 1:**
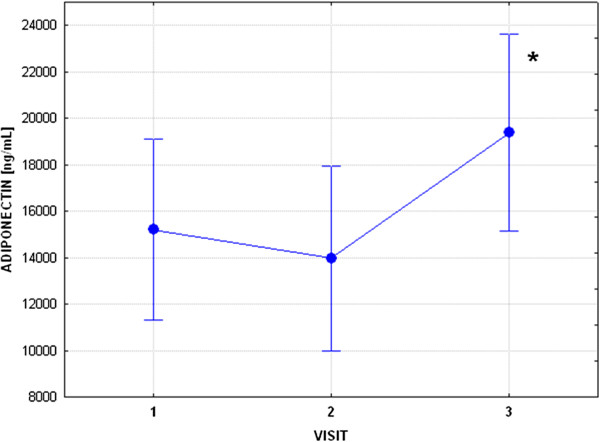
**Mean adiponectin concentrations and standard deviations in subjects treated with radioactive iodine, i.e., before radioiodine therapy (RIT), visit 1 (V1), seven days post RIT, visit 2 (V2), and two-three months post RIT, visit 3 (V3).** Statistical significance is marked by “*” (V1 vs V3, p<0.05).

**Figure 2:**
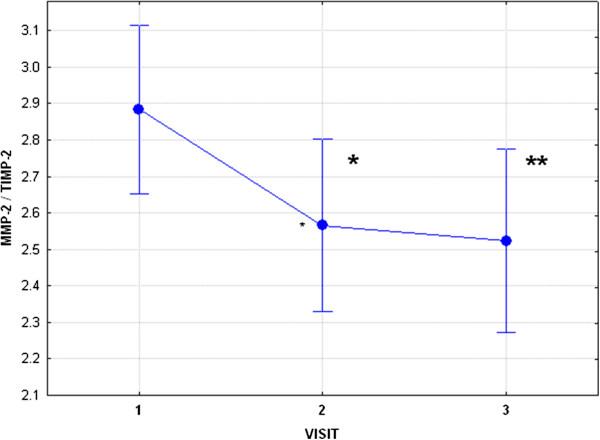
**Mean MMP-2/TIMP2 ratio and standard deviations in subjects treated with radioactive iodine, i.e., before radioiodine therapy (RIT), visit 1 (V1), seven days post RIT, visit 2 (V2), and two-three months post RIT, visit 3 (V3).** Statistical significance is marked by “*” (V1 vs V2, p<0.05), and by “**” (V1 vs V3, p<0.01).

Results of the study are presented in Tables 1, 2, 3. 4, Figure 1 and Figure 2. As expected, in patients from Group 1, there was a marked fall in FT_4_ and FT_3_. There was, however, no significant change in concentrations of MMP-2, MMP-9, TIMP-1, TIMP-2 and adiponectin (Table 1).

**Table 1:** **Descriptive statistics for the measurement results *“before” *and “*after” *thiamazole therapy (Group 1; n=15)**

	**mean**	**median**	**SD**	**min**	**max**	**p-value**
**MMP-2 – before** [ng/ml]	671.7	668.0	73.4	551.8	824.0	*0.78*
**MMP-2 – after** [ng/ml]	616.7	612.0	76.2	446.0	733.0	
**MMP-9- before** [ng/ml]	928.0	880.0	464.8	300.0	1960.0	*0.93*
**MMP-9 – after** [ng/ml]	1238.7	1200.0	421.7	650.0	1890.0	
**TIMP1- before** [ng/ml]	374.7	390.0	109.8	70.0	500.0	*0.83*
**TIMP1- after** [ng/ml]	382.0	370.0	70.8	280.0	510.0	
**TIMP2- before** [ng/ml]	207.7	205.0	42.8	150.0	280.0	*0.18*
**TIMP2- after** [ng/ml]	179.7	185.0	38.2	105.0	245.0	
**Adiponectin – before** [ng/ml]	30807	31190	12996	8450	52090	*0.36*
**Adiponectin – after** [ng/ml]	32755	34490	16638	2800	56100	
**TSH - before** [mIU/l]*	0.007	0.005	0.003	0.005	0.01	***0.013***
**TSH – after** [mIU/l]	1.064	0.03	1.74	0.0	5.77	
**FT**_**3**_**– before** [pg/ml]*	8.74	7.53	4.79	4.02	16.5	***0.0010***
**FT**_**3**_**– after** [pg/ml]	3.49	3.54	2.40	0.74	4.8	
**FT**_**4**_**– before** [ng/dl]*	4.48	2.21	1.01	7.84	32.5	***0.0010***
**FT**_**4**_**– after** [ng/dl]	1.02	1.07	0.44	0.34	1.7	

p-value for the Mann-Whitney’s test for a comparison of pairs of measurement results *“before”* and “*after”*; SD – standard deviation; p – level of significance.

*Reference ranges: TSH - 0.27-4.2 mIU/l,

FT_4_ - 0.93-1.7 ng/dl (1 ng/dl = 1 pmol/l x 12.87),

FT_3_ - 2.6-4.4 pg/ml (1 pg/ml = 1 pmol/l x 1.54).

**Table 2:** **Descriptive statistics for the levels of TSH, FT**_**4**_**and FT**_**3**_**before radioiodine administration and at three months after radioiodine administration (Friedman ANOVA test), Group 2, n=20; SD – standard deviation; SEM – standard error of mean; p – level of significance**

	**Mean**	**Med**	**Min**	**Max**	**SD**	**SEM**	***p-value***
**TSH** [mIU/l]	0.122	0.027	0.004	0.877	0.230	0.060	***0.00012***
	1.74	0.82	0.03	7.89	2.27	0.59	
**FT**_**4**_ [pmol/l]	24.4	18.8	13.3	72.5	15.4	4.0	***0.014***
	14.7	13.9	3.9	58.3	10.8	2.4	
**FT**_**3**_ [pmol/l]	10.0	7.7	4.1	24.0	5.6	1.4	***0.016***
	6.1	5.1	2.2	25.8	4.8	1.1	

Reference ranges: TSH - 0.4-4.0 mIU/l,

FT_4_ - 11.5-22.7 pmol/l (1 pmol/l x 0.0777 = ng/dl),

FT_3_ - 2.76-6.45 pmol/l (1 pmol/l x 0.6493 = pg/ml).

**Table 3:** Descriptive statistics for the levels of matrix metalloproteinases (MMP–2, MMP–9), their inhibitors (TIMP-1, TIMP-2) and adiponectin at three time points, i.e. before radioiodine treatment (RIT), visit 1 (V1), seven days post RIT, visit 2 (V2), and two-three months post RIT, visit 3 (V3), p-value for the Friedman ANOVA test, Group 2, n=20

	**Visits**	**Mean**	**Med**	**Min**	**Max**	**SD**	**SEM**	***p-value***
**MMP-2** [ng/ml]	V1	356	358.0	243.9	488.0	73.2	16.8	*0.24*
	V2	349	348.8	221.8	470.3	63.3	14.9	
	V3	362	373.1	283.2	449.2	54.0	13.5	
**MMP-9** [ng/ml]	V1	647	500.0	220.0	1390.0	343.8	78.9	*0.56*
	V2	584	500.0	180.0	1790.0	378.5	89.2	
	V3	628	655.0	180.0	1020.0	261.9	65.5	
**TIMP-1** [ng/ml]	V1	157	160.0	70.0	260.0	52.7	12.1	*0.063*
	V2	173	170.0	100.0	260.0	40.8	9.6	
	V3	174	175.0	110.0	220.0	29.7	7.4	
**TIMP-2** [ng/ml]	V1	129	120.0	75.0	285.0	45.3	10.4	***0.012***
	V2	139	135.0	95.0	215.0	36.3	8.6	
	V3	149	137.5	90.0	220.0	38.3	9.6	
**Adiponectin** [ng/ml]	V1	15201	11890.0	3780.0	37970.0	8860.0	2032.6	***0.0004***
	V2	13966	13020.0	3740.0	24990.0	6432.4	1516.1	
	V3	19373	17190.0	8010.0	38550.0	8657.4	2164.4	

p-value for the Friedman ANOVA test, Group 2, n=20.

**Table 4:** Descriptive statistics for the levels of MMP-9/TIMP-1 and MMP-2/TIMP-2 ratio at three time points, i.e. before radioiodine treatment (RIT), visit 1 (V1), seven days post RIT, visit 2 (V2), and two-three months post RIT, visit 3 (V3), p-value for the Friedman ANNOVA test, Group 2, n=20

	**Visit**	**Mean**	**Med**	**Min**	**Max**	**SD**	**SEM**	***p-value***
**MMP-9/TIMP-1** [ng/ml/ng/ml]	V1	4.58	3.14	1.90	14.71	3.36	0.77	*0.22*
	V2	3.53	2.95	0.90	8.95	2.18	0.51	
	V3	3.62	3.81	1.06	6.2	1.49	0.37	
**MMP-2/TIMP-2** [ng/ml/ng/ml]	V1	2.88	2.85	1.59	3.73	0.54	0.13	***0.03***
	V2	2.57	2.56	1.80	3.20	0.39	0.09	
	V3	2.52	2.45	1.91	3.6	0.53	0.13	

p-value for the Friedman ANOVA test, Group 2, n=20.

In patients from Group 2, there was also a significant fall in FT_4_ and FT_3_, as well as an increase in TSH from V1 to V3 (Table 2). There was no significant change in MMP-2, MMP-9, TIMP-1 and adiponectin from V1 to V2, but there was an increase in TIMP-2 concentrations (p<0.05) (Table 3, Figure 1, Figure 2). Subsequently, however, there was an increase in serum concentrations of adiponectin, while concentrations of TIMP-2 still remained elevated in comparison to V1 (p<0.01), see Table 3, Figure 1 and Figure 2. Given that TIMP-2 is the principal inhibitor of MMP-2, while TIMP-1 is the principle inhibitor of MMP-9, we have endeavoured to assess whether there is a possibility of a change in concentrations of free (i.e. active) MMP-2 and MMP-9. For these reasons we have also assessed MMP-9/TIMP-1 and MMP-2/TIMP-2 ratio. Results of this analysis are presented in Table 4. There was a decrease in MMP-2/TIMP-2 ration (p<0.05), confirming the possibility of a fall in free MMP-2 concentrations at about 3 months after radioiodine administration.

## Discussion

To the best of our knowledge, this is the first study where concentrations of matrix metalloproteinases and their inhibitors were assessed before and after treatment of thyrotoxicosis. The lack of significant change in concentrations of MMPs indicates that matrix metalloproteinases are unlikely to contribute to cardiovascular morbidity and mortality associated with thyrotoxicosis. To some extent, this thesis is also corroborated by lack of any significant change in concentrations of adiponectin before and after normalization of thyroid function. Our results are in keeping with the study of Iglesias et al. [16] who reported lack of any significant difference in serum adiponectin in hyperthyroid (n=20) vs euthyroid subjects (n=20). Also in the study of Chu et al. [17], the authors reported that hyperthyroidism-associated insulin resistance was not mediated by adiponectin levels. It should be mentioned, however, that there are studies suggestive of slightly higher adiponectin concentrations in hyperthyroid subjects, particularly with Graves’ disease [18,19], though in these studies concentrations of adiponectin have not been assessed in the same subjects, before and after treatment.

In contrast to subjects treated with thiamazole, there was a clear increase in adiponectin concentrations at about three months after radioiodine administration. As, to some extent this was also accompanied by a fall of FT_4_ and FT_3_ to reference values, thus a question arose whether some increase in adiponectin concentrations might be observed after longer period of observation in subjects treated with thiamazole. There is also a question whether the observed increase in adiponectin in subjects treated with radioiodine would be sustainable over a longer period. This issue, needs to be addressed by further research. Nevertheless, the observed increase in vasoprotective adiponectin is reassuring, given some recent controversies regarding cardiovascular safety of treatment with radioactive iodine. As mentioned above [6,7], adiponectin improves insulin sensitivity and exerts anti-atherosclerotic effects in blood vessels. Indeed, there are some studies [20] suggestive that the plasma leptin/adiponectin ratio predicts first cardiovascular event, at least in men. Furthermore, many cancer cell lines express adiponectin receptors, and adiponectin *in vitro* limits cell proliferation and induces apoptosis. Recent *in vitro* studies demonstrate the antiangiogenic and tumor growth-limiting properties of adiponectin [21]. There are also some studies that an association between malignancy and low adiponectin concentrations exists, for instance in the case of colorectal cancer [22]. We also noticed that even though there was no significant change in concentrations of matrix metalloproteinases (MMP-2 and MMP-9), there was some increase in TIMP-2 concentrations and a fall in MMP-2/TIMP-2 ratio that might reflect some decrease in concentrations of free, i.e., presumably active MMP-2, as TIMP-2 is the principal inhibitor of MMP-2, while TIMP-1 is the principal inhibitor of MMP-9 [23]. Therefore, our data support a notion expressed previously that treatment with radioactive iodine appears safe [24], i.e., an observation that was even applied to children treated with radioactive iodine for Graves’ disease [25]. It should be mentioned, however, that cardiovascular safety of radioactive iodine would also depend on meticulous follow-up of patients who undergo this treatment as efforts must be made to detect and to treat radioiodine-induced hypothyroidism. If this is not done properly, then hypothyroidism *per se* would increase a risk of subsequent cardiovascular disease [26-28]. It should also be mentioned that stable concentrations of matrix metalloproteinases were also reassuring in terms of potential risk of neoplastic disease. In this context, we noticed that large study of Ron et al. [29], based on a data from 35 593 subjects, failed to reveal an increase in cancer mortality in subjects treated with radioiodine.

In summary, our study demonstrates that radioiodine treatment of thyrotoxicosis does not alter serum MMP-2, MMP-9 or TIMP-1 concentrations, either acutely or after about three months of observation. Though such data alone are not enough to fully conclude upon the issue of cardiovascular safety of radioiodine administration, the lack of the above mentioned alterations of matrix metalloproteinases is at least reassuring. Furthermore, an increase in serum adiponectin might reflect favourable effects of radioiodine administration on cardiovascular and cancer risk factors, while an increase in TIMP-2 (principal MMP-2 inhibitor) might lead to a decrease in free MMP-2 concentrations.

## Competing interests

The authors declare that they have no competing interests.

## Authors’ contributions

AL designed and coordinated the study, and revised the text of manuscript, AB supervised the radioiodine therapy of patients and participated in acquisition of data, KCL participated in coordination of the study and drafted the manuscript, ES-J participated in acquisition of data, AM conceived the study and participated in design of manuscript. All authors have read and approved the final manuscript.
